# Supplementary material for: Chromothripsis during telomere crisis is independent of NHEJ, and consistent with a replicative origin
Source: Genome Res. 2019 May;29(5):737–49. doi: 10.1101/gr.240705.118 (PMC6499312; doi:10.1101/gr.240705.118)
Supplement: Supplemental Material [file supp_gr.240705.118_Supplemental_file_1.zip › contigs/annotated_contigs/DB83/contig.2.DB83_length_764_mean_cov_10.4057591623.docx]

**DB83_length_764_mean_cov_10.4057591623**

```
GCAAAGTGCAAATGCAACTATGGTAAATACCACCAGGGATGAGAATAGTCAGTTCACTTATCTCAAGTTAAATATGAGAGTAAAACTAC
 >chr2:78095031-78095435 - E=3e-228 p=1e-03
AATTTTAAGCCTAGAAAATACATGCTAAAGGCATATTAACTTGGAATTTGGAACTTACTCTATTGACTACATTTTTTTCTACATTGGAA

AAATCAGAAAAAAAAAGAAATGGAGAATTAAACATTATAGAAAACAAAAGAAGAGCAAAAATATGTTTTAATAGAAATTTTAAAAATAT

AGCACTTATCAAATTTGTTTCAACCATGACCCTGAATTTATTTTTGTTTCTTTAACTTAACTCATTGAAGAATGTGAAAGATCAATGGT

GTCTAAGTTTGATCAATAGAATAATATCAAAAATACTTTTTCACAAAT|TGTGA|AAGTTCCTGTTGTCAGTGCCAAGATTAACTGTGA
                                                      >chr2:78093546-78093901 - E=1e-19
CCAGAAACTCCCCGTGCCACTCACTGGCTGTTTGAAAGAAACATCTGACAGAGACTTGGCTGATTTTGGGTCTAGAGACCACCTAATCA
5
GGGCTCAACTATTTTGGCCAATCAGAAATGAACAAGTTTGAAACTTTCATTTGTGTAAGTGGACCTGGTTGACAACCTAGGTGAGAACT

TTCCCTATTTAAGCCAGTCCCTCCCTTTGTTCTGTGGAGAGCATTCTTTTATTTCTATTGAATGTTATGCCTCCCCAGTCAATGGATTA

TTTTATAATAGAAATTAAGCTTTCCATTTTTTCTCCACAGATCTCATGGCCTTT
```
